# Current landscape of social media use pertaining to glioblastoma by various stakeholders

**DOI:** 10.1093/noajnl/vdad039

**Published:** 2023-05-24

**Authors:** Mohammed Ali Alvi, Lior M Elkaim, Jordan J Levett, Alejandro Pando, Sabrina Roy, Nardin Samuel, Naif M Alotaibi, Gelareh Zadeh

**Affiliations:** Division of Neurosurgery, Toronto Western Hospital, University Health Network, Toronto, Ontario, Canada; Department of Neurology and Neurosurgery, McGill University, Montreal, Quebec, Canada; Department of Medicine, University of Montreal, Quebec, Canada; Department of Neurosurgery, Rutgers New Jersey Medical School, Newark, New Jersey, USA; Department of Laboratory Medicine and Pathobiology, University of Toronto, Toronto, Ontario, Canada; Division of Neurosurgery, Toronto Western Hospital, University Health Network, Toronto, Ontario, Canada; Department of Neurosurgery, National Neuroscience Institute, King Fahad Medical City, Riyadh, Saudi Arabia; Division of Neurosurgery, University of Toronto, Toronto, Ontario, Canada

**Keywords:** glioblastoma, GBM, social media, twitter

## Abstract

**Background:**

Given the potential for social media to allow widespread public engagement, its role in healthcare, including in cancer care as a support network, is garnering interest. To date, the use of social media in neuro-oncology has not been systematically explored. In the current manuscript, we sought to review Twitter use on glioblastoma among patients, caregivers, providers, researchers, and other stakeholders.

**Methods:**

The Twitter application programming interface (API) database was surveyed from inception to May 2022 to identify tweets about glioblastoma. Number of tweet likes, retweets, quotes, and total engagement were noted for each tweet. Geographic location, number of followers, and number of Tweets were noted for users. We also categorized Tweets based on their underlying themes. A natural language processing (NLP) algorithm was used to assign a polarity score, subjectivity score, and analysis label to each Tweet for sentiment analysis.

**Results:**

A total of 1690 unique tweets from 1000 accounts were included in our analyses. The frequency of tweets increased from 2013 and peaked in 2018. The most common category among users was MD/researchers (21.6%, *n* = 216), followed by Media/News (20%, *n* = 200) and Business (10.7%); patients or caregivers accounted for only 4.7% (*n* = 47) while medical centers, journals, and foundations accounted for 5.4%, 3.7%, and 2.1%. The most common subjects that Tweets covered included research (54%), followed by personal experience (18.2%) and raising awareness (14%). In terms of sentiment, 43.6% of Tweets were classified as positive, 41.6% as neutral, and 14.9% as negative; a subset analysis of “personal experience” tweets revealed a higher proportion of negative Tweets (31.5%) and less neutral tweets (25%). Only media (β = 8.4; 95% CI [4.4, 12.4]) and follower count (minimally) predicted higher levels of Tweet engagement.

**Conclusion:**

This comprehensive analysis of tweets on glioblastoma found that the academic community are the most common user group on Twitter. Sentiment analysis revealed that most negative tweets are related to personal experience. These analyses provide the basis for further work into supporting and developing the care of patients with glioblastoma.

**Key Points**

- MDs or researchers are the most common user group on Twitter discussing glioblastoma
- Patients and caregivers’ presence on Twitter is much less common compared to other neurosurgical pathologies
- Tweets containing embedded media (video or image) were associated with increased retweets, quotes, and likes

**Importance of the Study**

Given the importance of social media in engaging healthcare stakeholders, in the current manuscript, we sought to review Twitter use on glioblastoma among patients, caregivers, providers, researchers, and other stakeholders. We anticipate the social media use patterns identified in this study will be of broad interest to stakeholders involved in care of patients with glioblastoma including providers, payors, pharmaceutical and technological business and patient advocacy groups. We also believe that the results of this study can relevel recurring trends in communication related to glioblastoma and can inform strategies for support in patient care for individuals and families affected by this disease.

Glioblastoma is the most common and the most aggressive malignant primary brain tumor accounting for 48% of all malignant brain and CNS tumors and about 57% of the average yearly age-adjusted incidence rate of all neuroepithelial tumors.

In the last decade, discoveries, and major advancements in the treatment of glioblastoma have been made.^1^ Despite improvement in overall survival, recurrence remains a significant challenge. Due to the lack of a global standard of care for recurrent glioblastoma, the therapeutic choices are limited once the tumor recurs.^2^

Social support can help patients with illnesses cope with their disease. Social media platforms such as Twitter, Facebook, Instagram, and YouTube provide users access to an online community.^3^ Patients are increasingly using social media for both emotional support and educational goals related to their healthcare problems.^4–6^ Further, healthcare practitioners are also using social media to increase awareness, promote cutting-edge treatments and technologies, and share work. These features can improve the understanding of patient health conditions and enhance dialogue.^4^ Recent studies have made an effort to understand social media and evaluate its influence on academia.^4,5,7^ Importantly, these platforms have also been incorporated in “altmetrics,” such as assessment of dissemination and advocacy of medical and clinical research.^8^

Given the importance of social media in engaging healthcare stakeholders, in the current manuscript, the authors sought to detail the social media ecosystem surrounding glioblastoma from multiple perspectives, including patient or caregiver, physician or care team, media or news outlets and businesses. The results from this study can relevel recurring trends in communication related to glioblastoma and can inform strategies for support in patient care for individuals and families affected by this disease.

## Methods

### Search Strategy

We conducted a comprehensive search of the Twitter API database for Academic Research for all tweets pertaining to glioblastoma. The following search terms were used in isolation: “glioblastoma.” Abbreviations for glioblastoma, ie, “GBM” or “#GBM” were not searched as many Tweets including these terms were unrelated to glioblastoma. The entire Twitter database from inception to May 2022 (search date) was queried, and only English-language Tweets were included.

### Social Media Metrics and Data

For accounts, we eliminated duplicates, bots, those with fewer than 15 followers, and accounts with fewer than 10 tweets. Bots were identified by review of account username, description, and activity and were excluded if any of these self-identified the account as a bot. A random sample of 2000 tweets was then selected. These tweets were further evaluated through manual review of tweets, and only those directly pertinent to glioblastoma in humans were included for final analysis. Originating account location, number of followers, number of tweets, and year joined were among the data that were extracted. Each Twitter account was assigned a category based on its goal or purpose, public title, and user-submitted Twitter account descriptions. Based on an initial screening, and using a strategy outlined in a prior publication,^9^ we developed categories to describe the accounts: Foundation, Business, Journal, Patient or Caregiver, Support Group, Medical Center, News or Media Outlet, MD or Researcher, and Other.

Further collected information included each tweet’s number of likes, retweets, quotations and entire free text. Duplicate tweets were deleted. Two authors independently assessed and validated each extracted tweet (M.A.A, L.E). Only tweets that were deemed pertinent to glioblastoma in humans were included for analysis. Using a modified version of thematic analysis and the axial and coding techniques provided in a prior paper,^9^ each tweet was given a category for its theme. All tweets were evaluated by both researchers, who created codes for any repeating themes. Any discrepancies in categorization were discussed. The final topics of categories for each tweet included one of the following: Personal experiences, raising awareness, discussing research or publications, advertising, fundraising, or “other.”

### Statistical Analysis

All social media data, such as follower count, tweet count, tweet likes, and tweet retweets, were given descriptive statistics (median, IQR). Multivariable regression models were used to identify predictors of higher engagement. In line with previous social media studies, data were not normally distributed (tested using Shapiro-Wilk test). All statistical analyses were performed using R-4.1.3 statistical program with an alpha value set at 0.05.

### Sentiment Analysis

To process the Tweets for sentiment analysis, we used a natural language processing (NLP) Python module called Textblob.^10^ This NLP library rated text on a polarity and subjectivity scale using a lexicon-based method. To assess this data semantically, the program used a pre-defined lexicon of phrases that were labeled according to whether they were positive or negative. Sentences in the body of the Tweets underwent pre-processing to receive analysis labels, polarity scores, and subjectivity scores. The polarity score ranged from [−1,1], with −1 denoting negative feelings and 1 denoting good ones. The presence of negative or positive words determines the polarity score. Words like “great” or “best,” for instance, have a polarity score of 1, while “disgusting” or “awful” have a score of −1.

The subjectivity score ranged from [0, 1], with 1 denoting personal information and 0 denoting objective information. An overall negative, neutral, or positive analysis was indicated by an analysis score of 0, 0, or > 0, respectively. By combining the average semantic values from different words, tweets were graded.

### Ethical Consideration

All the Tweets used in this study were cross-sectional, archived, and unpaid for, taken directly from the Twitter API without any involvement from other social media users. There were no usernames included. As a result, the current study does not need institutional research board approval in accordance with the Canadian Tri-Council Policy Statement for Research because all of the data are readily accessible to the general public.^11^

## Results

### Account Analysis

**Figure 1.**
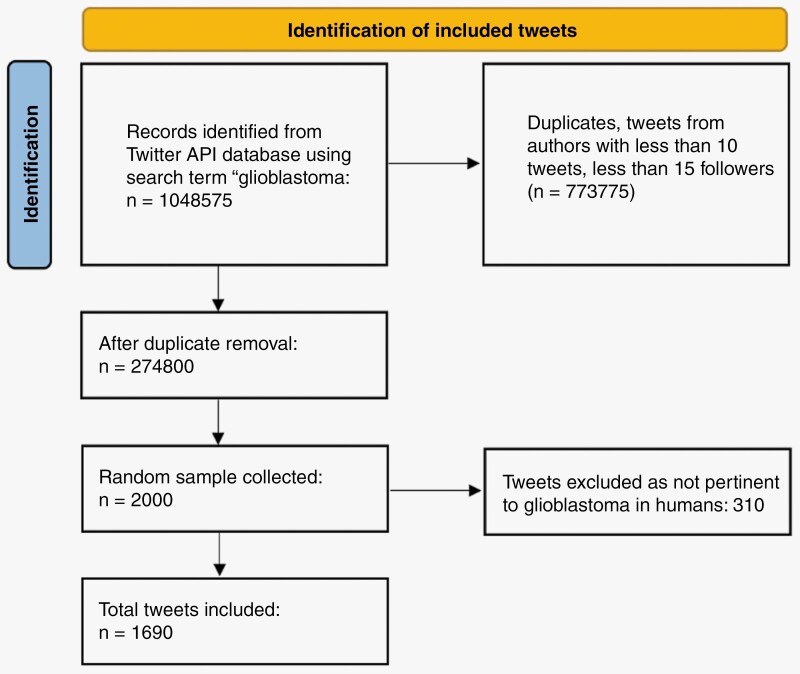
Flowchart detailing the inclusion of Tweets analyzed in the current manuscript.

Our search strategy identified 1048575 individual tweets mentioning glioblastoma from 2009 to May 2022. After duplicate Tweet removal, 274,800 tweets remained (Figure 1). After selection of a random sample of tweets, 1690 tweets from 1000 unique accounts meeting inclusion criteria were included. The median (IQR) number of followers for the accounts was 1240 (IQR: 390, 4176). The median number of tweets (IQR) per account was 12025 (IQR: 3302, 39021). The most common category among users was MD or researchers (21.6%, *n* = 216) followed by Media/News (20%, *n* = 200) and Business (10.7%); patients or caregivers accounted for only 4.7% (*n* = 47) while medical centers, journals, and foundations accounted for 5.4% (*n* = 54), 3.7% (*n* = 37), and 2.1% (*n* = 21). Table 1 provides a summary of included accounts. A 100 account sample from the “MD/Researcher” group found that 51% of accounts were directly related to neurosurgery or oncology (neurosurgeons, oncologists, neuropathologists, or academic experts) while 49% were other affiliated physicians and researchers..”

**Table 1.** Summary of Included Accounts

Number of Accounts		1000
Followers (median, IQR)	1240 (390, 4176)	
Tweets (median, IQR)	12,025 (3302, 39,021)	
Account Category	MD/Researcher	216 (21.6)
	Media/News	200 (20.0)
	Business	107 (10.7)
	Medical center	54(5.4)
	Patient/Caregiver	47 (4.7)
	Journal	37 (3.7)
	Support Group	28 (2.8)
	Foundation	21 (2.1)
	Podcast	13 (1.3)
	Support Group	28 (2.8)
	Other/unspecified	254 (25.4)

### Tweet Analysis

**Figure 2.**
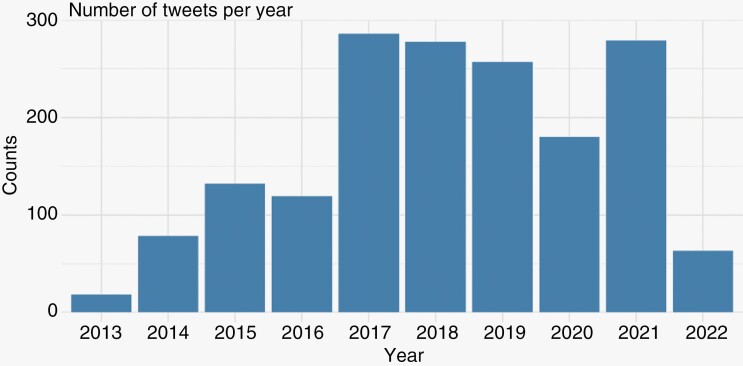
Figure demonstrating the number of Tweets per calendar year from the random sample of 1690 included tweets.

Tweet frequency climbed from 2013 (*n* = 18) to a peak in 2018 (*n* = 278), then decreased over the following 2 years (*n* = 257 and *n* = 180), before increasing once again in 2021 (Figure 2). Average engagement per tweet was 5.03 (SD) (32.20). Overall, 1364 included tweets (80.7) had a link, while 328 (19.4%) contained some type of media (video or image). Additionally, 622 (36.8%) of tweets featured a tag for another user or account, and 690 (40.8%) of tweets contained a hashtag. Table 2 provides a summary of tweet features.

**Table 2.** Summary of Included Tweet Characteristics

Number of Tweets		1690
Followers (median, IQR)		1240 (390, 4176)
Tweets (median, IQR)		12025 (3302, 39021)
Tweet Category (*N*, %)	Advertising	29 (1.7)
	Fundraising	62 (3.7)
	Other	94 (5.6)
	Personal experience	308 (18.2)
	Podcast	28 (1.7)
	Raising Awareness	257 (15.2)
	Research	912 (54.0)
Tweet year (*N*, %)	2013	18 (1.1)
	2014	78 (4.6)
	2015	132 (7.8)
	2016	119 (7.0)
	2017	286 (16.9)
	2018	278 (16.4)
	2019	257 (15.2)
	2020	180 (10.7)
	2021	279 (16.5)
	2022	63 (3.7)
Retweets (mean [*SD*])		1.07 (4.96)
Replies (mean [*SD*])		0.30 (1.62)
Likes (mean [*SD*])		3.54 (26.46)
Quote_count (mean [*SD*])		0.11 (0.58)
Total engagement (mean [*SD*])		5.03 (32.20)
Includes link (*N*, %)	No	326 (19.3)
	Yes	1364 (80.7)
Includes media (*N*, %)	No	1362 (80.6)
	Yes	328 (19.4)
Includes tagging (*N*, %)	No	1068 (63.2)
	Yes	622 (36.8)
Includes hashtag (*N*, %)	No	1000 (59.2)
	Yes	690 (40.8)

The most encountered tweet theme was “Research,” seen in 54% (*n* = 912) of tweets. These tweets usually discussed novel therapeutic strategies, or scientific findings from peer-reviewed journals. For example, one researcher tweeted “Check out our recent paper in @CellStemCell [link] which highlights a novel immunotherapeutic approach to target glioblastoma - a highly aggressive, adult brain cancer..” The next most common theme was personal experience, seen in 18.2% (*n* = 308) of cases. Many of these tweets focused on caregivers or acquaintances or family members reflecting on their experience witnessing their loved ones suffering from glioblastoma eg, “Having lost my mom in February to glioblastoma after a 14-month battle as well, my heart goes out to Senator John McCain, @MeghanMcCain, and their whole family during this incredibly difficult time. Cherish these last few weeks together and get some moments alone if you can.” Raising awareness (15.2%, *n* = 257) was the third most common theme. These tweets featured references to common questions related to glioblastoma, eg, “Glioblastoma: symptoms, treatments, how it develops. Got questions? Ask here. An oncologist will join us to answer them on [link] next..” Other themes found in our analysis included “advertising,” usually for conferences, journals, or medical centers, and “fundraising” and “podcast.”

On multivariable regression, tweets containing media (video or image) (β = 8.4; 95% CI [4.4, 12.4]) and follower count (β < 0.01; 95% CI [0.00006, 0.0001]) were the only factors associated with an increase in engagement metrics (retweets, quotes, and likes). The full multivariable model results can be found in Table 3.

**Table 3.** Results from Multivariable Regression Model for Predictors of User Engagement (sum of likes, retweets, replies, and quote-tweets)

	β	0.025 interval	0.975 interval	*P*-value
Intercept	0.5	−11.8	12.9	.93079
Fundraising	0.1	−14	14.1	.99345
Other	−1	−14.3	12.4	.88763
Personal Experience	1.5	−10.8	13.8	.81055
Podcast	0.7	−15.9	17.2	.93750
Raising awareness	−0.8	−13	11.5	.90294
Research	1.9	−9.9	13.6	.75354
Followers	0.00008	0.0006	0.0001	<.00001*
Media	8.4	4.4	12.4	.00004*
Tagging	2.5	−1	5.9	.16041
link	−0.2	−4.8	4.4	.94143
hashtag	1.5	−1.7	4.7	.34971

*Denotes statistical significance.

### Sentiment Analysis

In terms of sentiment, 43.6% of Tweets classified as “positive,” 41.6% as neutral, and 14.9% classified as negative. An example of a Tweet classified as “positive” is “Glioblastoma took the life my friend, [name]. He was by my side throughout my awkward middle school years and beyond. I am forever changed because I knew him. So happy to share that [link] is raising funds for a scholarship his deserving name.” Examples of negative Tweets included “my husband was only [age] when he died of glioblastoma. We fought hard for 17mo I miss him every day. So little funding goes toward this cancer yet [it’s] one of the deadliest. Thanks” and “I’m so very sorry. We also went through a glioblastoma diagnosis and lost my father as result.” Interestingly, when analyzing tweets that fall in the “personal experience” subgroup, we found a significantly higher proportion of negative Tweets (31.5%) and less neutral tweets (25%) (*P* < .001). We also performed multivariable logistic regression to analyze factors associated with” negative sentiment.” We found that the presence of a tag, link, or hashtag were predictive of negative sentiments.

## Discussion

In 2020, there were 3.8 billion daily active social media users. This represents half of the world’s population and 80% of all internet users. Social media, particularly Twitter, has emerged as a key platform for communication, information dissemination, and connections between individuals and groups in the medical field because of its accessibility, efficiency in reaching a wide range of people, and capacity to do so across geographies and time zones.^12^ In neurology and neurosurgery, glioblastoma is one of the deadliest pathologies. The current manuscript aims to describe the social media ecosystem for glioblastoma from a variety of perspectives, including those of the patient and caregiver, the physician and health care team, the media and news outlets, and industry.

### Tweet Themes

We found that the most common user category on Twitter was “MD or Researcher,” with most of these tweets featuring “research” related to glioblastoma (54%). Twitter has been the most commonly used platform for posts relating to research publications and initiatives due to its short character limit and real-time publishing possibilities.^13^ Most of the time, there were links to newly released patient resources, scientific discoveries, or published scientific studies. This is compatible with Twitter’s objective of facilitating the spread of new knowledge. This has been corroborated in previous studies investigating the use of twitter for other In a previous study by Murthy and Eldredge, which investigated the association of volume of tweets and region related factors, the authors used more than 90000 tweets related to cancer and found that a higher concentration of doctors and cancer centers were both associated with higher volume of cancer tweets.^14^ In another study investigating the use of twitter for messages related to cervical cancer, Teoh et al. found that healthcare providers were the most common group among those that tweeted from a personal account.^15^ Similarly, healthcare providers and organizations with medical backgrounds were among the most common group that provided content for melanoma on Twitter in a study by Gomaa et al.^16^

Neurosurgery and Neuro-oncology leadership in North America has increased its presence on social media platforms, including Twitter. The Congress of Neurologic Surgeons (CNS) Tumor Section launched its Twitter ­account in 2017, under “@NSTumorSection.” The account now has almost 5000 followers, and posts can reach up to 3900 people.^17^ The account also often shares new research published in reputed neuro-oncology journals, new guidelines, and seminars and other events pertaining to neuro-oncology.

Another organization using Twitter is the Brain Cancer Quality of Life Collaborative (BCQoLC),^18^ which includes individuals with brain tumors, care partners, researchers, palliative care and neuro-oncology professionals, advocacy organization leaders, and payer representatives. The organization uses multi-stakeholder panels and design-thinking workshops as engagement tools to determine research priorities linked to quality of life and palliative care for people with brain tumors, including glioblastoma. They have previously used “tweet chats” to better understand stakeholder perspectives outside of the panel and to validate potential ideas. In these tweet-chats, Twitter users participate using an agreed-upon hashtag (#BTSM for brain tumor social media). Recent data have shown that aggregated data from such chats can be analyzed using sentiment analysis and qualitative thematic analysis, such as those performed in this manuscript.^19–21^

The next most common category of Twitter users was news or media. The majority of these were online news sources, posting links to news articles about the diagnosis and demise of notable individuals such as American politicians (such as Ted Kennedy, John McCain, and Beau Biden) and entertainment or sports figures appearing frequently in the news (ie, reporters, singers, or athletes). In fact, the sharp increase in the number of tweets in 2018 may be attributed to the death of Senator John McCain. Interestingly, this “celebrity” phenomenon” has been observed in other studies as well. For example, in the study by Teoh et al., the authors found that the tweets made by actress Cara Delevigne for the hashtag #SmearforSmear campaign was found to have the most amount of likes and engagement.^15^

The next category of users was business. These included pharmaceutical companies and other commercial businesses promoting novel advances in therapeutics and drugs, often containing links to news articles regarding breakthroughs. Direct-to-consumer advertising (DTCA) by pharmaceutical and other therapeutic companies is an increasingly common yet hotly contested practice.^22–24^ It is critical to keep track of the information that customers encounter while looking for drug information on popular social media platforms in addition to the official social media accounts of pharmaceutical companies. Researchers have shown that customer evaluations and testimonials can be convincing. Pharmaceutical companies’ marketing expenditures now include a larger portion of online promotional efforts or eDTCA, and more businesses are using social media for marketing.^22,25^

Patient or caregivers only accounted for 4.7% of accounts. This contrasts with some of our other work focusing on other pathologies like cervical myelopathy and the use of DBS, where patient or caregiver experiences accounted for a substantial proportion of analyzed tweets. This may be due to the abysmal prognosis associated with glioblastoma, rendering most patients unable to sustain a quality of life, or even the time or will to engage with social media.

### Engagement

We also analyzed engagement metrics for each tweet and found that tweets containing embedded media (video or image) were associated increased retweets, quotes, and likes. Dialogues in the form of tweet chat or “tweetorials” related to brain tumors can benefit greatly from the knowledge and experience of medical professionals. The spread of fact-based information depends on public participation in these discussions. Healthcare-related hashtags are tracked by businesses like symplur,^26^ which can provide medical practitioners access to popular ones. Finally, experts advise broad use of hashtags and media while producing material on Twitter, considering the considerable association between media usage and audience size.^27–29^

### Sentiment Analysis

As previously discussed, advanced analytic tools such as NLP for performing sentiment and thematic analyses have increasingly been used to study social media data.^30–32^ Sentiment analysis looks for opinions and feelings conveyed in free-text natural language, both positive and negative. Researchers can ascertain how social media users feel about particular subjects or medical treatments in this way. Sentiment analysis has been utilized in the healthcare industry to determine how Twitter users feel about a variety of topics. In studies on social media pertaining to neurosurgical diseases, formal sentiment analysis utilizing NLP has, however, been comparatively underutilized. In our analyses, 43.6% of Tweets were classified as “positive,” 41.6% as neutral, and 14.9% classified as negative. When analyzing tweets that fall only in the “personal experience” subgroup, we found a significantly higher proportion of negative Tweets (31.5%) compared to less neutral tweets (25%). This may be because most of these tweets often referred to a living or deceased relative, acquaintance or friend with glioblastoma.

### Strengths and Limitations

To the best of our knowledge, this is the first manuscript to perform a comprehensive analysis of twitter use pertaining to glioblastoma, including a sentiment or thematic analysis of tweets.

This study has several limitations. Importantly, we did not search other social media platforms like Facebook or Instagram. Facebook may provide a space where patients feel more comfortable discussing their experiences living with glioblastoma. We only analyzed Twitter data, due to the convenience of the API. Future studies could analyze other social media platforms to further glean perspectives of patients living with glioblastoma. Further, it is possible that some relevant tweets related to glioblastoma were missed by our search strategy. However, our search strategy was kept broad, and we initially collected over one-million Twitter posts mentioning glioblastoma. We also acknowledge that a significant proportion (roughly 25%) of users were classified as “other.” This number is higher than previous social media studies, and may represent a more diverse social media userbase for glioblastoma. Finally, social media trends tend to change rapidly, and the results in this study may not be as accurate in several years. Still, we tried to provide a detailed analysis of the social media landscape up to 2022.

## Conclusion

This comprehensive analysis of tweets on glioblastoma found that MDs or researchers are the most common user group on Twitter. Further, patients and caregivers’ presence on Twitter is much less common compared to other neurosurgical pathologies. Substantial Twitter presence was also noted among businesses and media or news outlets. Sentiment analysis revealed that most negative tweets were those relating to personal experience. We anticipate the social media use patterns identified in this study will be of broad interest to stakeholders involved in care of patients with glioblastoma including providers, payors, pharmaceutical and technological business and patient advocacy groups.

## References

[1] Aldoghachi AF , Aldoghachi AF, Breyne K, Ling KH, Cheah PS. Recent advances in the therapeutic strategies of glioblastoma multiforme. Neuroscience. 2022;491:240–270. 35395355 10.1016/j.neuroscience.2022.03.030

[2] Pitz MW , Desai A, Grossman SA, Blakeley JO. Tissue concentration of systemically administered antineoplastic agents in human brain tumors. J Neurooncol. 2011;104(3):629–638. 21400119 10.1007/s11060-011-0564-y PMC4020433

[3] Alotaibi NM , Badhiwala JH, Nassiri F, et al. The current use of social media in neurosurgery. World Neurosurg. 2016;88:619–624.e7. 26585734 10.1016/j.wneu.2015.11.011

[4] Smailhodzic E , Hooijsma W, Boonstra A, Langley DJ. Social media use in healthcare: a systematic review of effects on patients and on their relationship with healthcare professionals. BMC Health Serv Res. 206;16(1):1–14. 27562728 10.1186/s12913-016-1691-0 PMC5000484

[5] Yakar F , Jacobs R, Agarwal N. The current usage of Instagram in neurosurgery. Interdiscip Neurosurg. 2020;19:100553.

[6] Perrin A , Anderson M. Share of U.S. adults using social media, including Facebook, is mostly unchanged since 2018. Pew Research Center. Published April 10, 2019. https://www.pewresearch.org/fact-tank/2019/04/10/share-of-u-s-adults-using-social-media-including-facebook-is-mostly-unchanged-since-2018. Accessed July 22, 2022.

[7] Saxena RC , Lehmann AE, Hight AE, et al. Social media utilization in the cochlear implant community. J Am Acad Audiol. 2015;26(2):197–204. 25690778 10.3766/jaaa.26.2.8 PMC4487612

[8] Lee G , Choi AD, Michos ED. Social media as a means to disseminate and advocate cardiovascular research: why, how, and best practices. Curr Cardiol Rev. 2021;17(2):122–128. 31729303 10.2174/1573403X15666191113151325 PMC8226195

[9] Meng Y , Elkaim L, Wang J, et al. Social media in epilepsy: a quantitative and qualitative analysis. Epilepsy Behav. 2017;71(Pt A):79–84. 28554148 10.1016/j.yebeh.2017.04.033

[10] Loria. textblob Documentation. Release 015. https://media.readthedocs.org/pdf/textblob/latest/textblob.pdf

[11] Tri-Council Policy Statement: Ethical Conduct for Research Involving Humans – TCPS 2 (2018). Published April 1, 2019. https://ethics.gc.ca/eng/policy-politique_tcps2-eptc2_2018.html. Accessed July 22, 2022.

[12] Shlobin NA , Hoffman SC, Clark JR, Hopkins BS, Kesavabhotla K, Dahdaleh NS. Social media in neurosurgery: a systematic review. World Neurosurg. 2021;149:38–50. 33556595 10.1016/j.wneu.2021.01.135

[13] Haustein S , Bowman TD, Holmberg K, et al. Tweets as impact indicators: examining the implications of automated “bot” accounts on Twitter. J Assoc Inf Sci Technol. 2016;67(1):232–238.

[14] Murthy D , Eldredge M. Who tweets about cancer? An analysis of cancer-related tweets in the USA. Digit Health. 2016;2:2055207616657670. 29942562 10.1177/2055207616657670 PMC6001277

[15] Teoh D , Shaikh R, Vogel RI, et al. A cross-sectional review of cervical cancer messages on twitter during cervical cancer awareness month. J Low Genit Tract Dis. 201;22(1):8–12. 29271850 10.1097/LGT.0000000000000363 PMC5745036

[16] Gomaa BT , Walsh-Buhi ER, Funk RJ. Understanding melanoma talk on twitter: the lessons learned and missed opportunities. Int J Environ Res Public Health. 2022;19(18):11284. 36141558 10.3390/ijerph191811284 PMC9517519

[17] Membership & Social Media Update. Tumor News. Published December 3, 2020. https://newsletters.aans.org/tumor/tumor-news-fall-winter-2020/membership-social-media-update/. Accessed August 9, 2022.

[18] Brain Cancer Quality of Life Collaborative. Brain Cancer Quality of Life Collaborative. http://www.braincancerqol.org. Accessed August 9, 2022.

[19] Cutshall NR , Kwan BM, Salmi L, Lum HD. “It Makes People Uneasy, but It’s Necessary. #BTSM”: using twitter to explore advance care planning among brain tumor stakeholders. J Palliat Med. 2020;23(1):121–124. 31170019 10.1089/jpm.2019.0077 PMC6931910

[20] Bolderston W , Woznitza W. Twitter journal clubs and continuing professional development: an analysis of a# MedRadJClub tweet chat. Radiography. https://www.sciencedirect.com/science/article/pii/S107881741730161X 10.1016/j.radi.2017.09.005 29306372

[21] Litchman ML , Snider C, Edelman LS, Wawrzynski SE, Gee PM. Diabetes online community user perceptions of successful aging with diabetes: analysis of a #DSMA Tweet chat (Preprint). doi:10.2196/preprints.10176 PMC6716433 31518231

[22] Ventola CL. Direct-to-consumer pharmaceutical advertising: therapeutic or toxic? P T. 2011;36(10):669–684. 22346300 PMC3278148

[23] Deshpande A , Menon A, Perri M 3rd, Zinkhan G. Direct-to-consumer advertising and its utility in health care decision making: a consumer perspective. J Health Commun. 2004;9(6):499–513. 15764449 10.1080/10810730490523197

[24] Krezmien E , Wanzer MB, Servoss T, LaBelle S. The role of direct-to-consumer pharmaceutical advertisements and individual differences in getting people to talk to physicians. J Health Commun. 2011;16(8):831–848. 21512934 10.1080/10810730.2011.561909

[25] Gibson S. Regulating direct-to-consumer advertising of prescription drugs in the digital age. Laws. 2014;3(3):410–438.

[26] Katz MS , Anderson PF, Thompson MA, et al. Organizing online health content: developing hashtag collections for healthier internet-based people and communities. JCO Clin Cancer Inform. 209;3:1–10. 10.1200/CCI.18.00124 31251658

[27] Bibault JE , Katz MS, Motwani S. Social media for radiation oncologists: a practical primer. Adv Radiat Oncol. 2017;2(3):277–280. 29114592 10.1016/j.adro.2017.04.009 PMC5605316

[28] Hamidi N , Karmur B, Sperrazza S, et al. Guidelines for optimal utilization of social media for brain tumor stakeholders. J Neurosurg. 2022;136(2):335–342. 34298513 10.3171/2020.11.JNS203226

[29] Linzey JR , Graffeo CS, Wang JZ, Haider AS, Alotaibi NM. Neurosurgery and the rise of academic social media: what neurosurgeons should know. J Neurosurg. 208;129(4):1–5. 10.3171/2018.2.JNS172817 29979122

[30] Social media analytics market with COVID- 19 impact analysis, by component, analytics type, application (sales and marketing management, and competitive intelligence), deployment mode, organization size, vertical, and region - global forecast to 2026. Published November 5, 2021. https://marketpublishers.com/report/media-entertainment/social_media/global-social-media-analytics-market-by-components-data-sources-users.html. Accessed August 9, 2022.

[31] Yang L , Li Y, Wang J, Sherratt RS. Sentiment analysis for E-commerce product reviews in Chinese based on sentiment lexicon and deep learning. IEEE Access. 2020;8:23522–23530.

[32] Kumar A , Khan SU, Kalra A. COVID-19 pandemic: a sentiment analysis: a short review of the emotional effects produced by social media posts during this global crisis. Eur Heart J. 2020;41(39):3782–3783. 32678890 10.1093/eurheartj/ehaa597 PMC7454503

